# Role of Heterogeneous Reactions in the Atmospheric Oxidizing Capacity in Island Environments

**DOI:** 10.1021/acs.est.4c11647

**Published:** 2025-01-24

**Authors:** Chaoyang Xue, Hui Chen, Max R. McGillen, Hang Su, Yafang Cheng, Jörg Kleffmann, Guo Li, Mathieu Cazaunau, Aurélie Colomb, Jean Sciare, Langley DeWitt, Nicolas Marchand, Roland Sarda-Esteve, Jean-Eudes Petit, Alexandre Kukui

**Affiliations:** † Laboratoire de Physique et Chimie de l’Environnement et de l’Espace (LPC2E), CNRS−Université Orléans−CNES, Orléans Cedex 2 45071, France; ‡ Max Planck Institute for Chemistry, Mainz 55128, Germany; § Institut de Combustion, Aérothermique, Réactivité Environnement (ICARE), CNRS, Orléans Cedex 2 45071, France; ∥ School of Environmental and Chemical Engineering, Shanghai University, Shanghai 200444, China; ⊥ Physical and Theoretical Chemistry, University of Wuppertal, Wuppertal 42119, Germany; # Univ Paris Est Creteil and Université Paris Cité, CNRS, LISA, Créteil F-94010, France; ¶ Laboratoire de Météorologie Physique (LaMP), Observatoire de Physique du Globe de Clermont-Ferrand, Université Clermont-Auvergne, CNRS, UMR 6016, Clermont-Ferrand 63000, France; ∇ Laboratoire des Sciences du Climat et de l’Environnement, Orme des Merisiers, Gif-sur-Yvette 91190, France; ○ Climate and Atmosphere Research Center, The Cyprus Institute, Nicosia 2417, Cyprus; ⧫ Aix Marseille University, CNRS, LCE, Marseille 13007, France

**Keywords:** nitrous acid, heterogeneous reactions, NO_2_ conversion, nitrate photolysis, island environments

## Abstract

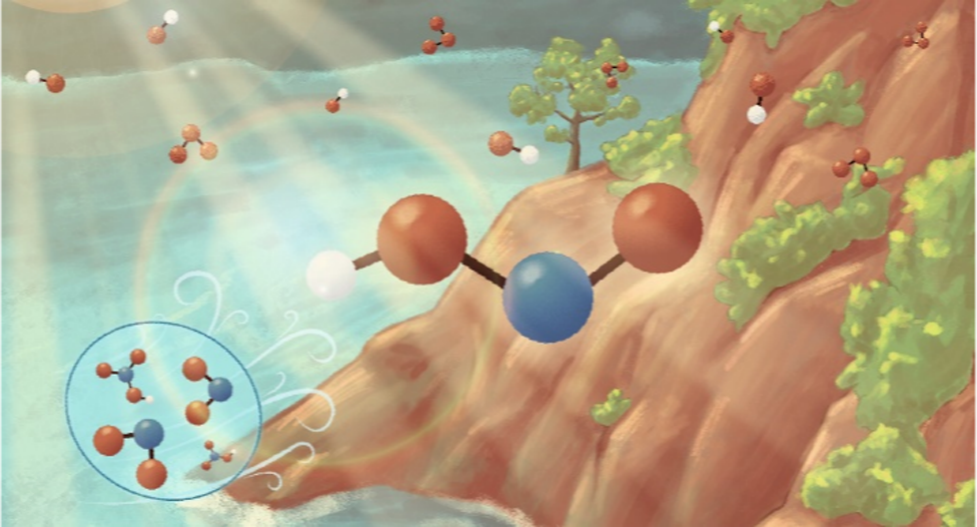

The source of nitrous acid (HONO) and its importance in island or marine environments are poorly understood. Herein, based on comprehensive field measurements at a hilltop on Corsica Island, we find an inverse diel variation of HONO with higher concentrations during daytime. Night-time HONO budget analysis indicates significant HONO formation during air mass transport along the hillside. In the daytime, although photosensitized NO_2_ uptake on the ground and NO + OH make considerable contributions (26% and 5%, respectively), a large part of HONO formation (67%, 320 pptv h^–1^) still cannot be explained with state-of-the-art parametrization. Nevertheless, photosensitized heterogeneous NO_2_ reactions are likely to account for the missing source, due to underestimation of the source by typical parametrizations at low NO_2_ levels. Furthermore, we demonstrate a significant role of HONO formation as a OH primary source at this island site, with a OH production rate exceeding one-fourth of that of O_3_ photolysis. Our findings underscore a potential role of heterogeneous surface reactions in the oxidizing capacity of the island environments.

## Abstract 2

Heterogeneous reactions on the island surface contribute significantly to daytime HONO formation and affect the atmospheric oxidizing capacity in island environments.

## 1 Introduction

While the atmospheric chemistry related to nitrous acid (HONO) has been studied for more than four decades,^1^ many uncertainties remain regarding its formation. It is important to understand how HONO is formed because of its significant impact on the oxidizing capacity of the lower troposphere by producing hydroxyl radical (OH) and follow-up impact on air pollution and climate.^2,3^ Hence, to identify major HONO sources, many field campaigns were conducted worldwide, most focusing on continental regions.^2,4,5^ However, only a few measurements have been targeted at the marine atmosphere where OH levels significantly affect the lifetime of greenhouse gases such as methane,^6^ indicating the necessity of studying OH sources in marine environments. Besides a few studies using aircraft/ship platforms,^7,8^ measurements on islands (or coastal areas) can also provide insights into marine atmospheric chemistry.^9−14^

Compared to high-NO_*x*_ environments (e.g., urban regions) where HONO formation and its impact have been well established, HONO over islands is poorly studied and it typically shows a different diel variation, i.e., higher levels in the day but lower levels at night.^9−11^ On the one hand, this indicates the non-negligible contribution of HONO to OH over the island and the surrounding ocean, while the contribution over continental regions is already well established. Furthermore, this may provide insights into chemical processes or boundary layer dynamics driving island HONO formation, which need to be further explored by field measurements. Notably, different HONO formation paths have been proposed for HONO formation at several island/coastal sites. In Cyprus, Meusel et al.^12^ observed higher HONO levels under low-RH than high-RH conditions, which was attributed to possible soil emissions based on laboratory experiments. At the Cape Verde CVAO site, Crilley et al.^11^ obtained a daytime minimum for ocean-derived HONO of 0.23 pptv, which was much lower than their measured HONO. Their results suggest a potential role of particulate nitrate (pNO_3_) photolysis but a minor role of the ocean surface in HONO formation, which was different from a previous study in a more polluted marine atmosphere.^13^ In Bermuda, Zhu et al.^14^ observed higher levels of HONO and HONO/NO_2_ in land cases than in sea cases but similar levels of pNO_3_ in both cases, indicating the important impacts of the land surface rather than pNO_3_ on HONO formation. Moreover, the HONO production unaccounted by NO_*x*_ or pNO_3_-related processes showed a significant positive correlation with daily average HNO_3_, suggesting photolysis of adsorbed HNO_3_ on the land surface as the possible source.^14^ Therefore, the understanding of HONO formation mechanisms over islands is still poorly understood. Comprehensive field measurements are needed to address the discrepancy in understanding HONO formation in island environments and its potential impact on the marine atmosphere.

In this study, within the framework of ChArMEx (the Chemistry and Aerosol Mediterranean Experiment), we conducted an extensive range of atmospheric measurements on Corsica. These measurements encompass HONO, various other trace gases, atmospheric radicals, and aerosol size and composition. The comprehensive data set allows a detailed analysis of the HONO budget. Combined with state-of-the-art HONO source parametrizations and box model simulations, we provide new insights into HONO chemistry in island environments: (1) HONO may contribute significantly to daytime OH production and (2) heterogeneous NO_2_ reactions are expected to dominate the missing HONO source.

## 2 Method

### 2.1 ChArMEx Campaign

**Figure 1:**
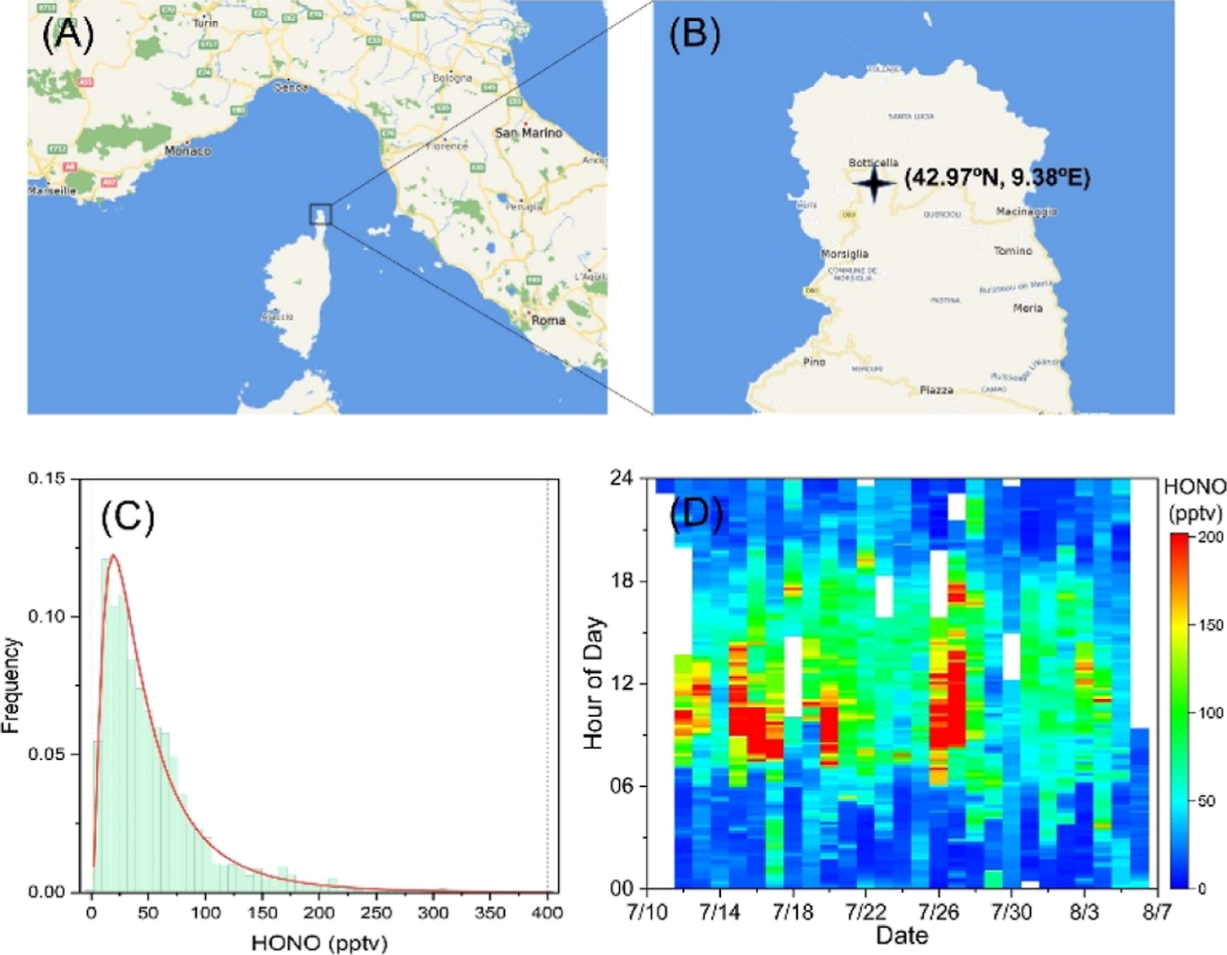
(A) Location of Corsica Island (Map copyright © 2022 Microsoft); (B) location of the measurement site (black cross); (C) histogram of the measured HONO data; and (D) time series of 10 min average HONO measurements during the ChArMEx campaign.

Data used in this study were obtained during the ChArMEx SOP2 (special observation period-2, https://mistrals.sedoo.fr/ChArMEx/, last access: 24 May 2023) field campaign, which was conducted in July and August 2013.^15,16^ Comprehensive measurements were conducted on the top of a hill (altitude: ∼530 m above sea level) in Corsica, France (42.97°N, 9.38°E, Figure 1A,B). It is about 6.0, 4.5, and 2.5 km from the sea in the east, north, and west directions, respectively.

### 2.2 Instrumentation

Table S1 shows the instruments providing major measurements used in this study. The instruments were placed in several containers with a distance of less than 50 m between each other. Briefly, HONO was measured by a wet chemical method (LOPAP-03, QUMA GmbH, Germany),^17^ which has been validated in a smog chamber and the ambient atmosphere by intercomparison with the DOAS technique.^18^ One potential interference (and/or HONO source) from the hydrolysis of nitrosyl chloride (ClNO) is discussed in Section 1 in the Supporting Information. The external sampling unit of LOPAP was installed on the top of a container (2.5 m above the ground level). Zero calibration was automatically conducted 2–3 times per day through sampling synthetic air (N_2_/O_2_ = 4:1). Liquid calibration was regularly carried out by a diluted nitrite solution in the sampling reagent under zero air. The measurement uncertainty was 10%, and the detection limit was a few parts per trillion (pptv) during this campaign. OH radicals were measured by a CIMS with detection limits of 5.0·10^5^ and 2.0·10^5^ molecules cm^–3^ for daytime and night-time measurements.^16,19^ Aerosol number size distributions in the ranges of 109–496 and 542–19,500 nm were measured by an SMPS TSI 3080 associated with a CPC TSI 3010 and an aerodynamic particle sizer APS TSI 3321, respectively. Aerosol composition and gas-phase HNO_3_/HCl were quantified by an Aerodyne HR-ToF-AMS and a wet denuder-ion chromatography (WAD-IC) system,^20^ respectively. O_3_ was measured by a commercial analyzer based on UV absorption (Thermo Fisher Scientific, Model 49i). Meteorology and J values were measured by an autometaphysical station and a spectral radiometer (Meteorologie Consult GmbH 6007), respectively. NO and NO_2_ were measured by the ECO PHYSICS CraNO_*x*_ II instrument, with a detection limit of ∼30 pptv. The NO_2_ channel of the CraNO_*x*_ II instrument is equipped with a photolytic blue light converter, which uses near-UV light to convert NO_2_ to NO before being detected, largely reducing interference from other odd nitrogen (NO_y_) species on the NO_2_ measurements.

### 2.3 Pseudo-Steady-State Approach

During the daytime, the relaxation of HONO concentration to pseudo-steady-state (PSS) conditions is reached on a scale of the HONO lifetime (τ(HONO), e.g., 10 min at noon, Figure S1).^6,21^ Therefore, for a homogeneously mixed atmosphere, such as the marine atmosphere, forest atmosphere, rural sites, etc., the PSS approach is typically used for daytime HONO analysis.^21,22^ In island regions, the underlying surface switches between land and ocean, which may significantly affect the composition of the corresponding boundary layer air masses due to variations of surface emissions, heterogeneous processes, and mixing. Therefore, when the contact time of the observed air mass with the land surface (t_land_) is shorter than that of τ(HONO), PSS conditions may not be achieved. For the daytime HONO budget analysis, data obtained during days with t_land_<τ(HONO) were filtered out to guarantee the validity of the PSS approach. In this study, t_land_ is estimated based on the wind speed (WS) and the distance to the sea in this wind direction, as demonstrated in detail by Kukui et al.^16^ As shown in Figure S1D, the calculated t_land_ is generally higher than τ(HONO), with exceptions mainly on 14, 23, 24, 25, 29, and 30 July and 6 August 2013 (therefore defined as t_land_ < τ(HONO) days).

### 2.4 Equations for the HONO Budget

During the day, when PSS is achieved, the HONO budget can be expressed by the following equation
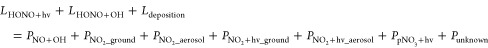
1

In this equation, *L*_HONO+hv_, *L*_HONO+OH_, and *L*_deposition_ represent the HONO loss through photolysis, reaction with OH, and deposition, respectively. *P*_NO+OH_, *P*_NO2_ground_, *P*_NO2_aerosol_, *P*_NO2+hv_ground_, *P*_NO2+hv_aerosol_, *P*_pNO3+hv_, and *P*_unknown_ denote the HONO production through the gas-phase reaction of NO + OH, NO_2_ uptake on the ground surface, NO_2_ uptake on the aerosol surface, photosensitized NO_2_ uptake on the ground surface, photosensitized NO_2_ uptake on the aerosol surface, particulate nitrate photolysis, and unknown sources, respectively. As shown in Figure S2, the observed NO and NO_2_ values were generally lower than 0.4 and 1.0 ppbv, respectively, with only several sharp and short spikes possibly resulting from car exhaust or occasional local anthropogenic emissions. Biomass burning (BB) emissions could be an important or dominant HONO source in relatively fresh plumes in the downwind areas of the BB locations.^23−25^ At the Corsica site, potential BB events were not often and were not large during the measurement period (see Figure S3). Moreover, the majority of air mass originated from the ocean rather than from the downwind areas of the potential BB evens (Figure S5A). Therefore, direct HONO emissions from combustion processes and photolysis of gas-phase nitro compounds^26^ are expected to be of minor importance at this site and have been excluded from consideration. Note that those processes may contribute to the observed HONO indirectly by contributing to NO_*x*_ on a regional scale. Unknown sources could result from soil emissions, acid displacement, photolysis of absorbed nitrate, and underestimated sources, as described in eq 1. Section 3.5 conducts detailed analyses and discussions to identify the major unknown source.

HONO sinks and sources can be parametrized according to the following equations^27,28^ with the used parameters explained in Table 1. Besides calculating HONO production and loss rates, the parametrization scheme can be further used in box models to simulate HONO concentrations (see Text S1 in the Supporting Information). Note that the uptake on the aerosol surface is not included in the parametrization as a HONO sink due to its low contribution during daytime.^29,30^
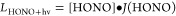
2
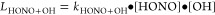
3
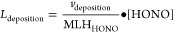
4
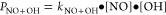
5
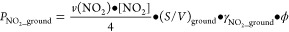
6
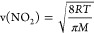
7
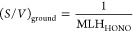
8
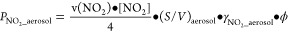
9
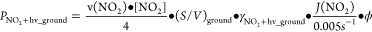
10
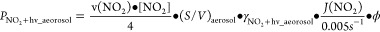
11
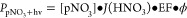
12

**Table 1:** Parameters Used in the HONO Source/Sink Equations^a^

parameter	meaning	value (range)	references
*J*(HONO)	HONO photolysis rate	measured, in s^–1^	
*k*_HONO+OH_	HONO + OH reaction rate coefficient	6.0·10^–12^ cm3 molecule^–1^ s^–1^	(1)
*v*_deposition_	deposition velocity of HONO	0.5 (0.1–2) cm s^–1^^b^	(2)
MLH_HONO_	mixing layer height for HONO	50 (10–200) m^c^	(3)
*k*_NO+OH_	NO + OH reaction rate coefficient	9.8·10^–12^ cm^3^ molecule^–1^ s^–1^	(1)
*v*(NO_2_)	mean NO_2_ molecular speed	calculated by eq-7, in m s^–1^	
*R*	ideal gas constant	8.314 kg m^2^ s^–2^ mol K	
*T*	temperature	measured, in K	
*M*	NO_2_ molar mass	0.046 kg mol^–1^	
*S*/*V*	surface-to-volume ratio	calculated by eq-8^d^, in m^–1^	
SEF	surface enhancement factor^e^	5 or 2.7	
γ_NO2_ground_	NO_2_ uptake coefficient on ground surfaces	1.5·10^–6^ (<10^–6^–10^–5^)	(4)
(*S*/*V*)_aerosol_	aerosol surface density	measured, in m^–1^	
γ_NO2_aerosol_	NO_2_ uptake coefficient on aerosol surfaces	1.5·10^–6^ (1·10^–7^–1.6·10^–5^)	(5)
γ_NO2+hv_ground_	photosensitized NO_2_ uptake coefficient on ground surfaces	2·10^–5^ (4·10^–6^–5·10^–5^)	(6)
*J*(NO_2_)	NO_2_ photolysis rate	measured, in s^–1^	
γ_NO2+hv_aerosol_	photosensitized NO_2_ uptake coefficient on aerosol surfaces	2·10^–5^ (10^–6^–10^–3^)	(6)
*J*(HNO_3_)	HNO_3_ photolysis rate	measured	
EF	enhancement factor of pNO_3_ photolysis compared to J(HNO_3_)	7 (1–700)	(7)
ϕ	HONO yield	0.5 or 1^f^	

a Value and Range represent the used value in this study and the reported range in the literature. Reference 1: the IUPAC Task Group on Atmospheric Chemical Kinetic Data Evaluation, values at 298 K, see https://iupac.aeris-data.fr (last access: 17 August 2023); 2:^31−34^; 3:^28,33,35^; 4:^33,36−38^ and this study; 5:^33,36,37^; 6:^5,36,39−43^; and 7:^8,44−46^.

b A value (0.5 cm s^–1^) close to the lower limit is used because a lower MLH than boundary layer height (BLH) is used in the parametrization.

c MLH rather than marine BLH should be used here because measurements are conducted near the ground surface. Using the BLH would strongly underestimate the impact of near-ground processes like heterogeneous production and deposition losses; we note that a constant MLH used here may cause uncertainties in ground-derived HONO sources.

d Projected geometric surface area (1 m^2^ m^–2^) is used to simplify *S*_ground_/*V*; the use of geometric surface area results in higher uptake coefficients compared to laboratory data.

e Similar to previous studies, surface enhancement factor (SEF) is not considered in the model parametrization in the base scenario; however, Section 3.3 explains that SEF should be taken into account.

f Here, we applied HONO yields (ratio between HONO desorbed from and formed on the surface) of 0.5 for dark NO_2_ conversion and 1 for daytime photosensitized reactions.

## 3 Results and Discussion

### 3.1 Field Measurements

Figure S4 shows the meteorological conditions during this campaign. Ambient temperature was generally within the range of 18–30 °C, with exceptions reaching ∼30 °C during 26–28 July. Atmospheric relative humidity (RH) was around 60% in the daytime and above 90% at night. Low RH levels were observed typically along with low-speed or northern winds. Southwest was the dominant wind direction (Figure S5A), with wind speeds generally lower than 5 m s^–1^. Strong southwestern winds of >5 m s^–1^ were observed on July 23, 24, and 30.

The measured HONO varied in the range of 2–400 pptv (Figure 1C), with an average of 56 ± 51 pptv and a median of 41 pptv. In general, much higher HONO levels were observed during the day than at night. Daily peaks were normally between 100 and 200 pptv, occurring at around 10:00–14:00 (Figure 1D). High HONO levels above 90 pptv are observed only in some east- and northeast-originated air masses, which is similar to the distributions of high NO_*x*_ (east and northeast) but opposite to high pNO_3_ (southwest, Figure S5). In terms of average diel variation (Figure S6), HONO measured at this site is low and mostly stable (<30 pptv) at night and starts to increase after sunrise. It shows a peak of 127 pptv at 9:50, which is similar to that of NO_2_ variations (peak at 10:10, Figure S6). Similar reverse diel variation has also been observed at other sites, which is discussed in detail in Sections 3.2 and 3.7.2.

### 3.2 Comparison with Other Coastal/Island Measurements

**Figure 2:**
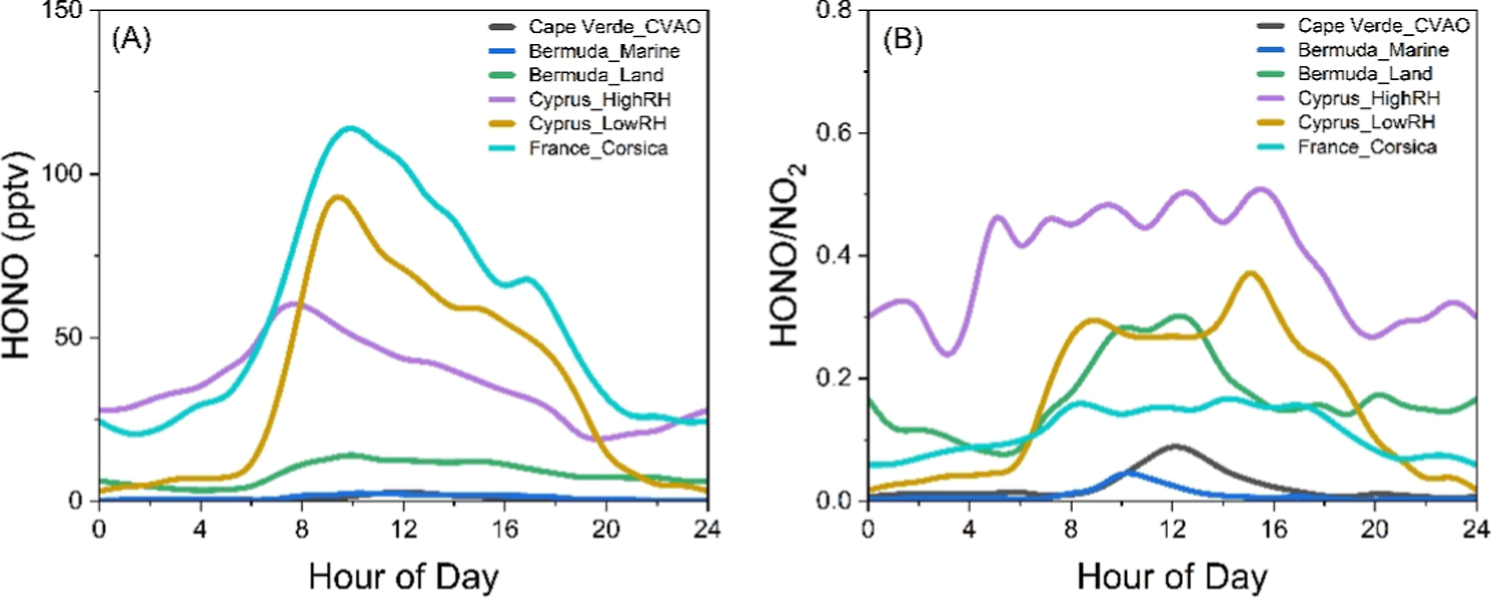
Diel variations of (A) HONO and (B) HONO/NO_2_ measured at four island sites worldwide: Cyprus (under low- and high-RH conditions), the Cape Verde Atmospheric Observatory (Cape Verde_CVAO), Bermuda (land and marine cases), and Corsica (France_Corsica).

Figure 2 shows the derivative variations of HONO and HONO/NO_2_ measured at four island sites worldwide (Table S2). The levels of HONO observed at those sites are different, varying from several to hundreds of pptv. However, their diurnal profiles behave similarly, with higher levels during the day than those at night. In particular, the France_Corsica site shows the highest daytime HONO levels among those sites and exhibits very similar levels and variations to those in Cyprus under low-RH conditions.

Similar to HONO, the HONO/NO_2_ ratios at all four of these sites perform a similar variation, with persistently higher levels during the day than at night. While the higher levels of daytime HONO/NO_2_ (10–50%) at those sites than most inland measurements (<20%)^28,47−50^ indicate the dominant role of secondary HONO formation, the significant difference in HONO/NO_2_ levels at the four island/coastal sites indicates the complexity of the relationship between HONO and its potential precursor NO_2_.

### 3.3 Night-Time HONO Budget

At night, the observed average HONO/NO_*x*_ ratio (9.3%) is much higher than that in vehicle exhaust (∼1%) potentially from pass-by vehicles (not frequent). While the HONO/NO_*x*_ ratio in BB emissions could be high,^46,51,52^ we have demonstrated that it is not a significant source at this site (see Section 2.4). The two findings above suggest the dominant role of secondary HONO formation.

Considering that our measurement site is on a small hill of ∼530 m altitude above sea level, the night-time mountain breeze (downslope wind) may affect the observations. Assuming that the night-time mountain breeze dominates the air mass flow, the observed HONO would remain close to the level in the upper boundary layer/residual layer. However, the observed night-time HONO at the Corsica site reached about 30 pptv, which is much higher than that for the upper boundary layer/residual layer (a few pptv),^46,51,52^ indicating that air mass arriving at the measurement site was not purely from those areas. Indeed, the upslope transport could be initialized by regional winds mainly from the southwest direction (distance to sea = 3.1 km) overwhelming the mountain breeze downslope air transport. And in fact, strong winds with wind speeds of 3–6 m s^–1^ were frequently observed during the nighttime (Figure S7A). Significant impact of upslope transport as well as the HONO production during the transport process on the observed HONO was also observed in our previous measurements at the summit of Mt. Tai (∼1500 m above sea level).^53^ As the hill at the Corsica site is much lower and smaller than that at Mt. Tai, a weaker mountain breeze can be expected at the Corsica site, making it easier to be overwhelmed by the regional winds.

The strong winds also lead to a *t*_land_ of 9–17 min, much smaller than the long HONO lifetime of around 2.7 h (against deposition and OH oxidization), indicating that the composition of the air arriving at the measurement site was influenced by its presence over both sea and the island. As the HONO concentration level of a few pptv typically observed for marine air mass^11,14^ is about 1 order of magnitude lower than that observed at the Corsica site (∼30 pptv), the HONO production during the upslope transport was likely the major contributor to the observed HONO at the Corsica site.

From 20:00 to 4:00, HONO levels are almost constant (), indicating the equality of sources and sinks, as shown in the equation below
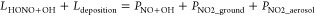
13

The average *L*_HONO+OH_, *L*_deposition_, *P*_NO+OH_, and *P*_NO2_aerosol_ during 20:00–4:00 are 6.9·10^–3^, 8.3, 0.02, and 0.01 pptv h^–1^, respectively, deriving a *P*_NO2_ground_ of 8.3 pptv h^–1^, indicating the dominant role of NO_2_ conversion on the ground surface in night-time HONO formation. Combined with eq 6, we obtain an uptake coefficient γ_NO2_ground_ of 7.3·10^–6^, which is at a relatively high level compared to previously reported ones (Table 1). This is likely due to the underestimation in (*S*/*V*)_ground_ as only the projected geometric surface area was used to describe HONO formation on ground surfaces (see eq 8). If, e.g., an SEF of 5 is considered, γ_NO2+h*v*_ground_ of 1.5·10^–6^ would also well describe the observations. This is quite reasonable considering that the true ground surface is not perfectly flat but instead exhibits vertical structures (such as stones, cracks, etc.) and significant porosity (e.g., large pores in the top layer of the soil, accessible for NO_2_ uptake), which could easily lead to an SEF of up to 5. During the daytime, the SEF is expected to be smaller for photochemistry as not all surfaces are exposed to solar radiation. Therefore, the SEF should be factored into the calculation of (*S*/*V*)_ground_, as shown in the following equation
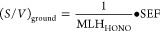
14

Figure S8 shows the sensitivity of γ_NO2_ground_ to the influencing factors, *v*_deposition_, MLH, and γ_NO2_aerosol_. Within the typical variation ranges of MLH or γ_NO2_aerosol_, γ_NO2_ground_ does not vary much around 1.5·10^–6^. In contrast, γ_NO2_ground_ is directly proportional to the used *v*_deposition_, leading to γ_NO2_ground_ variations by an order of magnitude for a typical *v*_deposition_ range. Other uncertainties could originate from the HONO yield as a HONO yield of 50% is used to represent all NO_2_-to-HONO conversion on the ground surface, including hydrolysis and, more importantly, the redox reactions.^33,38,54−56^

### 3.4 Daytime HONO Budget

**Figure 3:**
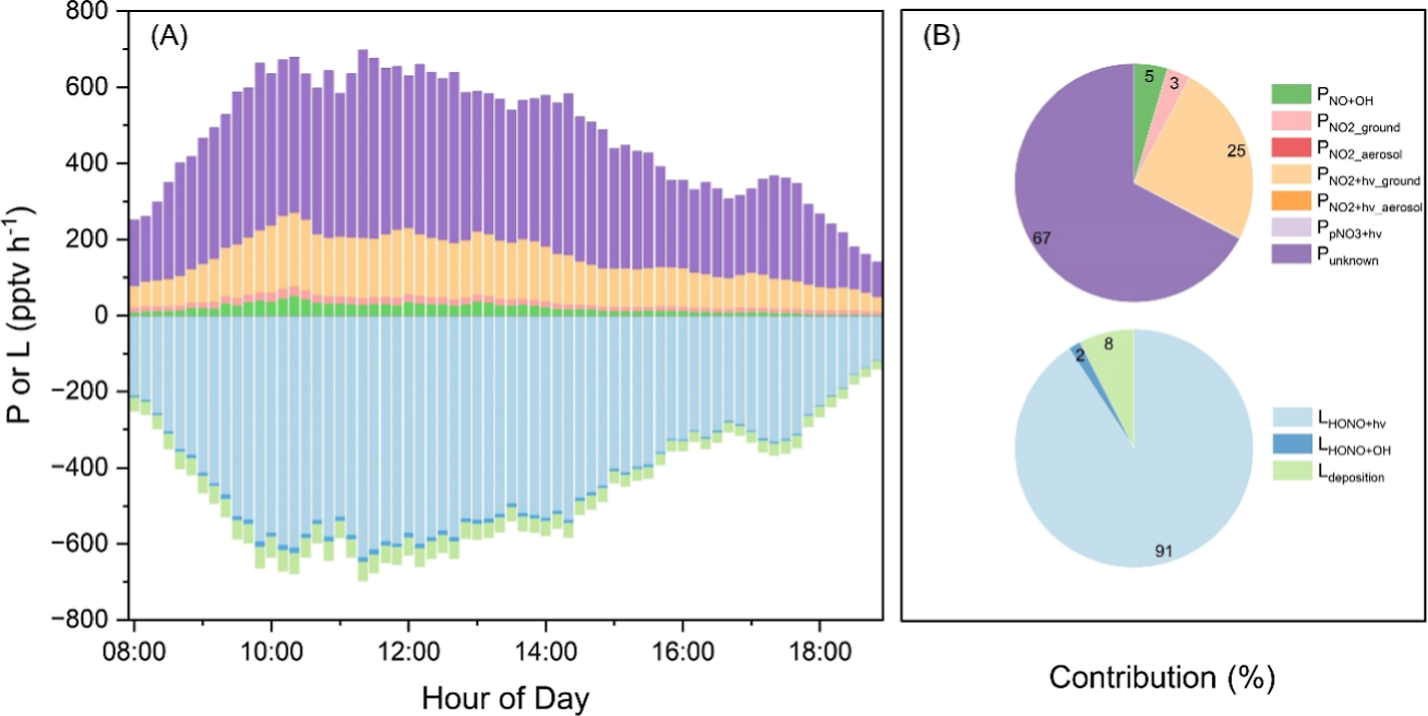
(A) Average diurnal profiles of daytime HONO formation (P) or loss (L) rates through different sources and sinks and (B) their relative contributions averaged over the daytime. *P*_unknown_ was calculated according to the pseudo-steady state (*P* = L). In panel B, labels are not shown if the percentage is less than 1%.

Figure 3 illustrates the average diurnal profiles of HONO formation and loss rates during the daytime. It is clear that photolysis dominates daytime HONO loss, with a contribution of 91%, while deposition and reaction with OH contribute the rest. Among the six sources, *P*_NO2+h*v*_ground_ makes the largest contribution of 25%, followed by *P*_NO+OH_ (5%) and *P*_NO2_ground_ (3%). Other three sources, including *P*_NO2+hv_aerosol_, *P*_NO2_aerosol_, and *P*_pNO3+hv_, contribute less than 1%. Even with the upper limits of γ_NO2+hv_aerosol_ and EF of 1.0·10^–3^ and 700, respectively, the contribution of *P*_NO2+hv_aerosol_ or *P*_pNO3+hv_ is still less than 8%.

With similar parametrizations as used here, HONO observations at inland sites could be generally explained.^27,28^ However, at this island site, the sum of the total HONO formation from the above-mentioned six sources is still much lower than the needed strength that can account for total HONO loss, which is in agreement with model results (see Text S2 in the Supporting Information for the description of the used model) that the modeled HONO concentration is lower than observations (Figure S9). This leads to a large unknown source (*P*_unknown_), varying from 90 to 490 pptv h^–1^, with an average of 318 ± 101 pptv h^–1^ (Figure 3). Interestingly, *P*_unknown_ shows asymmetric diurnal profiles compared to the diurnal profile of radiation, e.g., *J*(HONO) (Figure S10), with higher values in the morning compared to the afternoon, which is similar to the diurnal profiles of NO_2_ (Figure S6) and *P*_NO2+hv_ground_ (Figure 3).

### 3.5 Potential Sources to Explain *P*_unknown_

#### 3.5.1 NO_2_ Uptake on the Ground Surface

**Figure 4:**
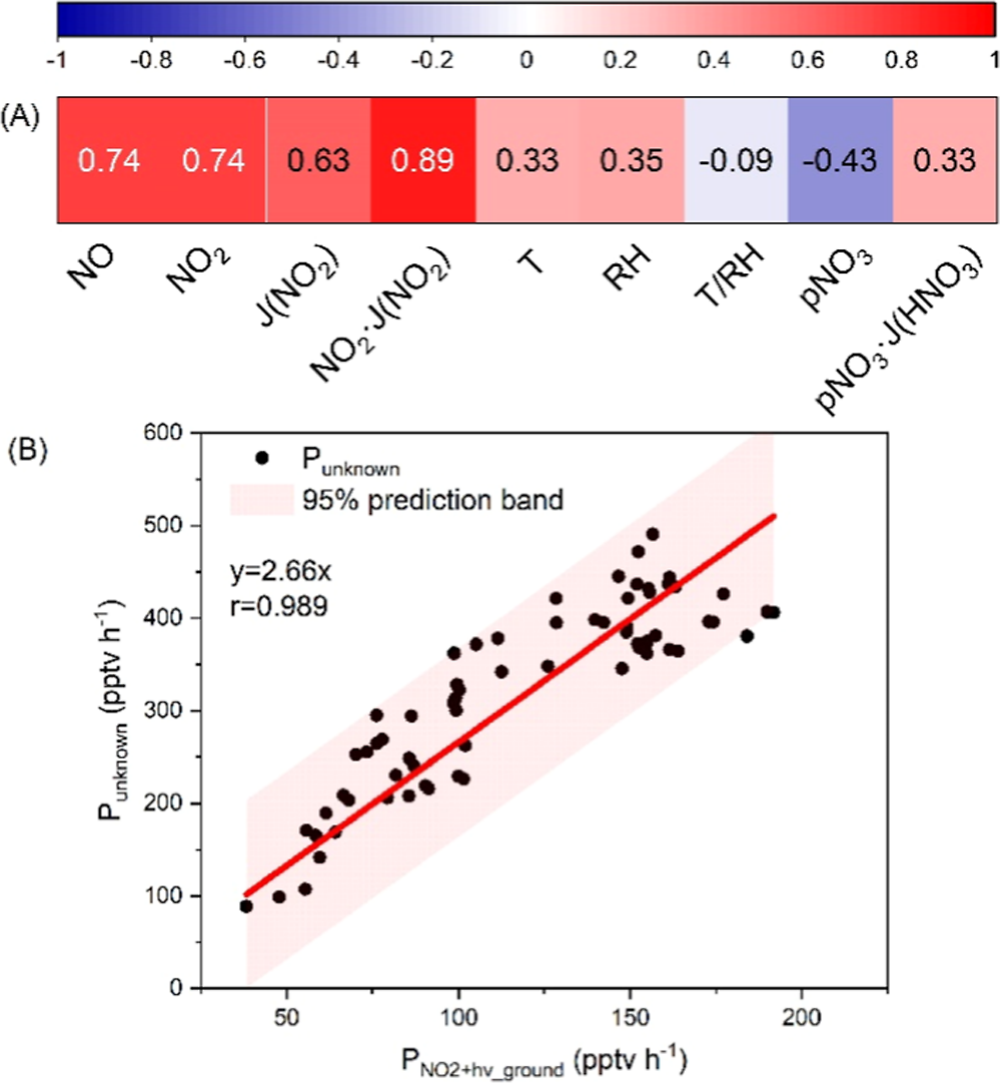
(A) Correlations between *P*_unknown_ and other parameters and (B) between *P*_unknown_ and *P*_NO2+h*v*_ground_. In panel (A), numbers and the color bar represent the correlation coefficient (*r*). *p*-Values for those correlations are <0.01 except for the correlation between *P*_unknown_ and T/RH.

With a laboratory-determined γ_NO2+hv_ground_ of 2·10^–5^ from Stemmler et al. (2006), *P*_NO2+h*v*_ground_ appears similar to *P*_unknown_ in variation but ∼2 times lower in strength (Figure 3A). Moreover, as shown in Figure 4, high correlations between *P*_unknown_ and NO_2_ (*r* = 0.74), between *P*_unknown_ and NO_2_·J(NO_2_) (*r* = 0.89), and between *P*_unknown_ and *P*_NO2+hv_ground_ (*r* = 0.989) indicate that a larger γ_NO2+h*v*_ground_ could help to explain the *P*_unknown_ reasonably. Then, in model Scenario 2, we use a larger γ_NO2+h*v*_ground_ of 5.3·10^–5^ and find that the model’s performance is significantly improved (Figure S9). The model can accurately predict the HONO peak and well predict the observed HONO diurnal profile. Moreover, laboratory studies suggest that γ_NO2+h*v*_ground_ exhibits an inverse relationship with the NO_2_ levels, showing higher values at lower NO_2_ concentrations. In Stemmler et al.,^39^ the γ_NO2+h*v*_ground_ of 2·10^–5^ was obtained at 20 ppbv NO_2_, much higher than the NO_2_ level at the Corsica site (daytime mean: 0.6 ppbv). Hence, a larger γ_NO2+h*v*_ground_ value can be expected for the Corsica site, and the enlarged *P*_NO2+hv_ground_ is likely to be the main source of *P*_unknown_. Alternatively, as discussed in Section 3.3, (*S*/*V*)_ground_ might be underestimated if SEF is not considered, leading to a similar effect as the underestimation in γ_NO2+h*v*_ground_. For instance, with an SEF of 2.66, the model predicts the same result as that with a γ_NO2+h*v*_ground_ of 5.3·10^–5^.

We should also note that while a γ_NO2+h*v*_ground_ of 5.3·10^–5^ or considering an SEF of 2.66 (or any combination where γ_NO2+h*v*_ground_ * SEF = 5.3·10^–5^) could explain the majority of *P*_unknown_, there is still a significant gap between predictions and observations during 4:00–10:00 (Figure S9). An even larger γ_NO2+h*v*_ground_ or SEF could help reduce the gap, but it is unlikely, as this adjustment would lead to discrepancies during other hours of the day. Moreover, the gap remains with additional consideration of potential primary HONO emissions (S2 + 1% NO_*x*_, Figure S9), which indicates uncertainties in the used parametrizations (e.g., variable MLH in the morning) or the coexistence of other sources.

#### 3.5.2 Biological Soil Emissions

Meusel et al.^9,12^ provided an indication of soil HONO emissions in Cyprus, where similar levels of unexplained *P*_unknown_ were observed. The HONO emission flux (*F*_HONO_) from Cyprus soil samples was determined under laboratory conditions. A maximum of 264 ng-N m^–2^ s^–1^ was observed under laboratory conditions and about 7.4 ng-N m^–2^ s^–1^ was estimated for field conditions, which could explain the observed *P*_unknown_ observed at both Corsica and Cyprus sites. Since both Corsica and Cyprus are Mediterranean islands with similar land cover and vegetation, biological soil HONO emissions could also contribute to the missing HONO source in the present study. Recent laboratory^57−59^ and field studies on fertilized soils^60−62^ find that *F*_HONO_ shows a positive correlation with temperature (*T*) and/or a negative correlation with soil water content or RH. Biological soil emissions become more important when nitrogen fertilizers are applied to agricultural soils,^61,63,64^ which is not the case for the soils in the surroundings of the Corsica site. At the Corsica site, *P*_unknown_ correlates poorly with T, RH, or T/RH (Figures 4 and S11). For instance, during 31 July–3 August, RH was at a low level (average: 45.6 ± 12.6%, Figure S4), but HONO during this period (average: 46.3 ± 33.3 pptv, Figure S2) is even slightly lower than the campaign average (HONO: 56.0 ± 50.8 pptv) with a higher average RH of 69.7 ± 20.0%. On the other hand, our observations are in good agreement with previous flux studies, in which HONO fluxes were attributed to photosensitized NO_2_ uptake on the ground surface^34,65−68^ or the photolysis of adsorbed nitrate.^69^ In contrast, laboratory experiments revealed the temperature and water content dependencies of biological soil HONO emissions, suggesting a noontime or early afternoon maximum of biological soil HONO emissions. For the uppermost soil layer that controls HONO fluxes, the soil temperature and water content should closely follow solar irradiance and inversely the atmospheric RH (Figure S11), respectively. Due to the difference in the observed and expected diurnal profiles, the biological soil source might coexist but is excluded as the main HONO source at the Corsica site.

#### 3.5.3 Photolysis of Adsorbed HNO_3_

During the campaign, HNO_3_ was measured by a WAD-IC instrument, and its average was 2.6 ± 1.0 μg m^–3^ (Figure S6). Using measured concentration and assuming a deposition velocity of 1 cm s^–1^, integrated deposition to the ground surface during one day of 2200 μg m^–2^ is calculated. Assuming that all those deposited HNO_3_ undergo photolysis with a recommended EF of 7^44^ (Table 1) and a HONO yield of 50%,^69−72^ the calculated daytime mean HONO production rate would be 68 pptv h^–1^, representing only a fifth of *P*_unknown_ (Figure S12). Using a higher EF of 25, the potential maximum HONO production from photolysis of adsorbed HNO_3_ would be comparable to observations. However, the significant time misalignment depicted in Figure S12 for the diurnal profiles suggests that photolysis of adsorbed HNO_3_ is not the major HONO source in this context.

#### 3.5.4 Other Sources

a) Photolysis of Particulate Nitrate

As discussed in Section 3.4, with an EF of 7, HONO formation from pNO_3_ photolysis (*P*_pNO3+hv_) makes a minor contribution to daytime HONO formation at the Corsica site. However, reported EF values are prone to large variations. On top of laboratory-reported ones ranging from a single digit to thousands,^70−74^ recent field constraints proposed that EF upper limits are not as high as in laboratory studies.^14,44,46,53^ Moreover, the poor correlations between *P*_unknown_ and pNO_3_ (*r* = −0.43, Figure 4) or between *P*_unknown_ and pNO_3_·*J*HNO_3_ (*r* = 0.33) also suggest a minor role of pNO_3_ in daytime HONO formation. This is also consistent with Friedrich et al., in which pNO_3_ photolysis made only minor contributions to ship-based HONO measurements over the Mediterranean Ocean.^7^

b) Acid Displacement

Night-time HONO deposition may constitute a daytime source through acid displacement.^75^ This process is limited by the amount of HONO deposited on the soil surface and the abundance of strong acids in the atmosphere.^75^ In Corsica, with a night-time HONO level of around 22 pptv, its deposition rate is about 1.6–16 pptv h^–1^ (considering night-time deposition velocities in the range 0.1–1 cm s^–1^). Assuming that all the deposited HONO at night (20:00–5:00) could be rereleased through acid replacement during the daytime (5:00–20:00), the average HONO production rate through this process would be 1–11 pptv h^–1^, which would have a negligible impact compared with the observed *P*_unknown_. In addition, since a maximum of the HNO_3_ concentration (the main strong acid for replacement) is observed in the afternoon (Figure S6), followed by consecutive deposition on ground surfaces, the expected diurnal profile of this source will be different from that of *P*_unkonwn_.

c) Sea-Surface-Derived Source

HONO over the Mediterranean Ocean was measured during the AQABA ship-based campaign in the summer of 2017 and the observed HONO was generally lower than 40 pptv (unpublished data from ref (7)), which is much lower than that at the Corsica site (Figure 1), indicating a minor impact of a sea-surface-derived source on HONO levels over island regions. Zhu et al.^14^ also observed a similar phenomenon: HONO in marine air was up to one magnitude lower than that in island air, which is reasonable considering the high pH (∼8) and the corresponding high effective solubility of HONO in seawater. Those two studies suggest that in situ HONO formation over the ocean will have only a minor impact on the observed HONO on the island. This is also justified considering that typical transport times from the sea surface to the measurement site were larger than the photolytic lifetime of HONO (Figure S1C).

### 3.6 Contribution to OH Initiation

**Figure 5:**
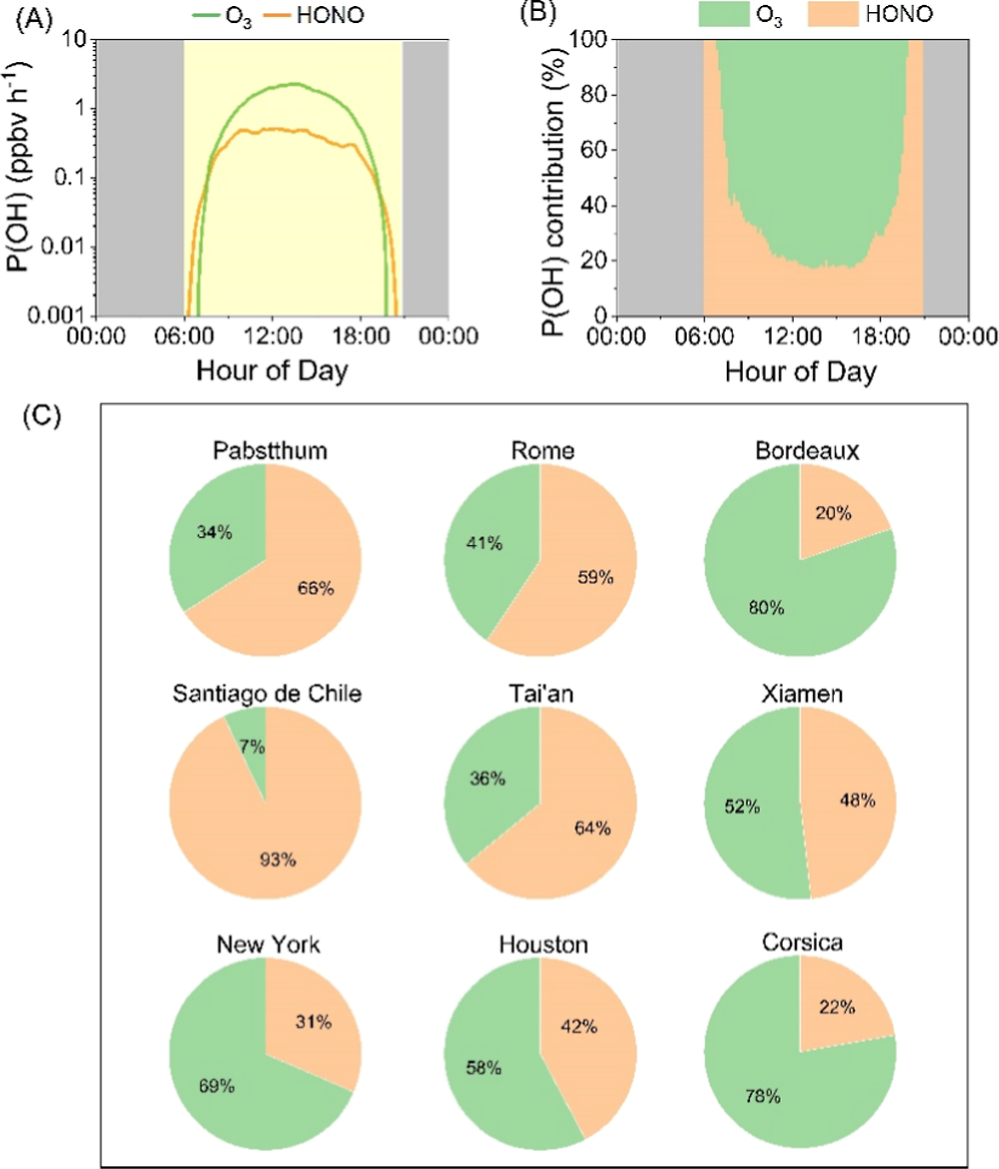
OH production rates from HONO and O_3_ photolysis. Average diurnal variations of (A) OH production rates; (B) relative contributions of the two OH sources; and (C) integrated daytime relative contribution of the two OH sources during summer in Pabstthum,^76^ Rome,^77^ Bordeaux,^78^ Santiago de Chile,^79^ Tai’an,^28^ Xiamen,^80^ New York,^81^ Houston,^82^ and Corsica (this study). Note that only two OH sources (HONO and O_3_ photolysis) were considered here for comparison.

Observed during the ChArMex campaign, the reverse diurnal variation of HONO constitutes a significant OH source during the daytime. As shown in Figure 5, compared to OH production from O_3_ photolysis, *P*(OH)_O3_, OH production from HONO, and *P*(OH)_HONO_ (gross production, estimated as HONO·*J*(HONO)), starts to rise earlier in the morning, initializing daytime photochemistry. During noontime, *P*(OH)_HONO_ stabilizes at around 0.5 ppbv h^–1^, more than one-quarter of *P*(OH)_O3_. The average relative contribution of *P*(OH)_HONO_ to *P*(OH)_HONO_ + *P*(OH)_O3_ is 39% (17–100%), even comparable to that observed in continental regions in summer (20–90%, Figure 5C), revealing the non-negligible role of HONO in OH production in island regions.

### 3.7 Atmospheric Implications

#### 3.7.1 Parameterization Schemes

Controversies in comprehending HONO formation may arise due to variations in used parametrization schemes. This study offers an overview of various HONO sources, sinks, and related parametrizations, which may serve as a crucial example for future investigations. The results obtained in this study have significant implications for advancing our understanding of HONO chemistry. While a comprehensive ChArMEx data set with a state-of-the-art HONO chemistry parametrization scheme was used, a large part of HONO formation (64%) still could not be explained, leading to a strong unknown source, *P*_unknown_, up to 470 pptv h^–1^. From excellent correlations of *P*_unknown_ with the product of NO_2_·*J*NO_2_ or *P*_unknown_ with *P*_NO2+hv_ground_, photosensitized NO_2_ reactions on the ground surface are likely to account for the *P*_unknown_ if a larger γ_NO2+h*v*_ground_ is used under low-NO_2_ conditions, as expected from γ_NO2+h*v*_ground_ dependencies on NO_2_ concentrations. Alternatively, model results can also be improved by considering ground surface roughness, highlighting the need for a more realistic parametrization of ground surfaces.

#### 3.7.2 Reverse Diurnal Profile

The “reverse” diurnal profile of HONO is not unique for island (Figure 2) or marine^8^ environments but is also observed in other continental sites, like mountain top,^81,83,84^ polar regions,^85−87^ remote background regions,^88^ and rural forest sites.^69^ The common factor for these locations is the low-NO_*x*_ environment, which is also characterized by even lower NO_*x*_ levels at night compared to daytime. Under these conditions, there is very low night-time HONO buildup resulting from direct combustion sources and/or secondary NO_2_ conversion. For the night-time heterogeneous NO_2_ conversion, first-order dependence was observed, i.e., the low night-time NO_2_ levels also imply slow heterogeneous HONO formation. In contrast, during the daytime, higher uptake coefficients of NO_2_ are observed for the photosensitized conversion of NO_2_, which are in addition inversely correlated with the NO_2_ concentration and may be thus even higher at low-NO_2_ conditions compared to lab conditions. Both slow night-time HONO formation and the increasing contribution of photosensitized reaction at low-NO_2_ levels contribute to the “atypical” daytime HONO maximum, as observed and discussed in this study. In contrast, when higher NO_2_ levels are present in the daytime and when NO_2_ in the daytime is lower compared to nighttime (typical for urban conditions), the HONO diurnal profile tends to follow a more “typical” variation, such as the urban coastal sites (e.g., coastal Weybourne, UK,^11^ and coastal Qingdao, China^13^) and many urban measurements. At those sites, night-time HONO levels increase due to direct combustion sources and much stronger secondary formation at high-NO_2_ levels, which hides smaller photosensitized HONO formation during daytime. Besides the heterogeneous NO_2_ conversion proposed in the present study, in very low-NO_*x*_ conditions (background sites, open ocean, etc.), additional sources like biological soil emissions^88^ and/or particulate nitrate photolysis^8,45^ could potentially play a role in the daytime HONO budget.

#### 3.7.3 Marine Atmospheric Oxidizing Capacity

As observed in this work, HONO formation is estimated to contribute significantly to OH production, comparable by strength to the photolysis of ozone. Under a low-NO_*x*_ condition such as the marine atmosphere, O_3_ production increases with primary OH production and NO concentration.^6^ Therefore, HONO photolysis can significantly enhance the production of O_3_ by contributing to the formation of both OH and NO. Moreover, longer lifetimes of NO_*x*_ and O_3_ than HONO allow more extended transport to the surrounding marine atmosphere. Here, islands provide surfaces for heterogeneously converting transported or ship-emitted NO_*x*_ to HONO. Both photolysis of HONO and secondarily formed O_3_ will potentially enhance the atmospheric oxidizing capacity and further affect the removal of greenhouse gases like methane (CH_4_). Hence, our findings suggest that islands may function as an “oxidizing pool”, potentially exerting an impact on the surrounding marine atmosphere. The results from the present study underscore the importance of incorporating HONO source parametrization into chemistry-transport and chemistry-climate models and quantifying the corresponding global impact. Furthermore, our results of HONO budget analysis indicate that ground surfaces still significantly affect island HONO measurements both during daytime and at night and thus those measurements may not accurately represent marine conditions. Accordingly, in situ measurements (ship-based or aircraft measurements) over the ocean are needed to better understand the formation of HONO in the marine atmosphere.

## Data Availability

The ChArMEx database is archived at https://mistrals.sedoo.fr/ChArMEx/under the corresponding data and publication policy (https://mistrals.sedoo.fr/ChArMEx/Data-Policy/ChArMEx_DataPolicy.pdf). HONO observations can also be obtained at 10.5281/zenodo.7558756.

## Supporting Information Available

Hydrolysis of nitrosyl chloride; box model; instruments providing measurements for this study; worldwide observations used for HONO intercomparison; daytime HONO, HONO/NO_2_, and NO_2_ plotted against the ratio of the contact time over land (*t*_land_) and the lifetime of HONO (τ(HONO)); time series of *t*_land_, τ(HONO), and *t*_land_/τ(HONO); time series of pNO_3_, NO_2_, NO, OH, J(HONO), and HONO measured during this campaign; active fire locations on Corsica Island during 11 July–7 August 2013; meteorological measurements (wind direction, WS, atmospheric pressure, solar irradiance, RH, and temperature) during the ChArMEx campaign; wind rose (WS against wind direction) and pollution rose plots (concentration or ratio against wind direction) for HONO, NO_2_, pNO_3_, NO_*x*_, and HONO/NO_2_ during this campaign; average diurnal profiles of HONO, J(HONO), OH, NO_2_, NO, particulate nitrate (pNO_3_), HNO_3_, aerosol surface density (*S*_aerosol_/*V*) and O_3_; night-time wind rose and pollution rose plots for HONO and HONO/NO_2_; sensitivities of γ_NO2_ground_ to *v*_deposition_, MLH, and γ_NO2_aerosol_; diurnal profiles of observed and model HONO; diurnal profiles of *P*_unknown_ at Corsica and Cyprus sites; average diurnal profiles of *P*_unknown_, atmospheric temperature (*T*), RH, and WS; and *P*_unknown_ and the calculated HONO production rate from the photolysis of adsorbed HNO_3_ (PDF)
