# Supplementary material for: Role of Heterogeneous Reactions in the Atmospheric Oxidizing Capacity in Island Environments
Source: Environ Sci Technol. 2025 Jan 24;59(6):3153–64. doi: 10.1021/acs.est.4c11647 (PMC11840933; doi:10.1021/acs.est.4c11647)
Supplement: Supplementary file 1 — es4c11647_si_001.pdf [file es4c11647_si_001.pdf]

Supporting Information for

Role of Heterogeneous Reactions in the Atmospheric Oxidizing Capacity in Island Environments

Chaoyang Xue[1,2,*], Hui Chen[3,4], Max R. McGillen[3], Hang Su[2], Yafang Cheng[2], Jörg Kleffmann[5], Guo Li[2], Mathieu Cazaunau[6], Aurélie Colomb[7], Jean Sciare[8,9], Langley DeWitt[10], Nicolas Marchand[10], Roland Sarda-Esteve[8], Jean-Eudes Petit[8], Alexandre Kukui[1,*]

[1]Laboratoire de Physique et Chimie de l'Environnement et de l'Espace (LPC2E), CNRS−Université Orléans−CNES, Orléans Cedex 2, 45071, France
[2]Max Planck Institute for Chemistry, Mainz, 55128, Germany
[3]Institut de Combustion, Aérothermique, Réactivité Environnement (ICARE), CNRS, Orléans Cedex 2, 45071, France
[4]School of Environmental and Chemical Engineering, Shanghai University, Shanghai 200444, China
[5]Physical and Theoretical Chemistry, University of Wuppertal, Wuppertal, 42119, Germany
[6]Univ Paris Est Creteil and Université Paris Cité, CNRS, LISA, F-94010 Créteil, France
[7]Université Clermont-Auvergne, CNRS, UMR 6016, Laboratoire de Météorologie Physique (LaMP), Observatoire de Physique du Globe de Clermont-Ferrand, Clermont-Ferrand 63000, France
[8]Laboratoire des Sciences du Climat et de l'Environnement, Orme des Merisiers, 91190 Gif-sur-Yvette, France
[9]Climate and Atmosphere Research Center, The Cyprus Institute, Nicosia, Cyprus
[10]Aix Marseille Univ, CNRS, LCE, Marseille, France

Correspondence:
Chaoyang Xue (ch.xue@mpic.de)
Alexandre Kukui (alexandre.kukui@cnrs-orleans.fr)

Contents

Number of Pages: 11

Number of Texts: 2

Number of Tables: 2

Number of Figures: 12

1 Supporting Texts 2
2 Tables 3
3 Figures 4
4 References 10

1 Supporting Texts

Text S1: Hydrolysis of Nitrosyl Chloride. The hydrolysis of nitrosyl chloride (ClNO) is reported as a HONO source[1,2], which may contribute to HONO formation in island environments and in addition might interfere with HONO measurements by wet chemical methods. ClNO can be produced in the reaction of NO_2 with sea salt aerosol. It is a low water-soluble gas, with Henry's law constant of higher than 0.05 mol L^{-1} atm^{-1} (lower than HONO under standard conditions, 47.5 mol L^{-1} atm^{-1})[3]. However, as Rubasinghege and Grassian (2012)[2] reported, ClNO is probably not formed under an RH level of more than 20%. At this site, the observed RH was always higher than 40%. Therefore, there might be a certain level of atmospheric ClNO in this marine region but is expected to be very low. Hence, ClNO should not show significant interference with wet chemical HONO measurements.

Text S2: Box Model. In addition to calculating the source strength (Section 2.4 in the main text), we employ a box model to test whether the comprehensive parameterization of HONO sources and sinks can reproduce the measured HONO concentrations. The Master Chemical Mechanism v3.3.1 operated on a MATLAB platform is used[4], which has been used in our previous study[5]. With previously described parameterization, the model is constrained by measurements of OH, NO, NO_2, pNO_3, $S_{aerosol}/V$, J values, and meteorological parameters, including temperature, pressure, and relative humidity. Regarding J(HONO), we used the absorption cross section evaluated by the IUPAC (https://iupac.aeris-data.fr/, last access: 30 September 2024). Two scenarios are designed: S1 as the base case with the above parameterization and S2 with an additionally enlarged $\gamma_{NO2+h\upsilon\_ground}$ or with the consideration of SEF.

2 Tables

Table S1. Instruments providing measurements for this study.

Species	Instrument
HONO	LOPAP, QUMA GmbH[6]
OH	CIMS[7]
NO, NO_2	CraNO_x II, ECO PHYSICS
Aerosol size (10.9 – 495.8 nm)	SMPS+CPC, TSI
Aerosol size (542 – 19480 nm)	SMPS+APS, TSI
Aerosol nitrate	HR-ToF-AMS, Aerodyne
HCl, HNO_3	Wet Denuder-IC[8]
O_3	Thermo Fisher Scientific, Model 49i
Meteorology	Auto meteo station
J values	MetCon 6007

Table S2. Summary of worldwide coastal HONO observations.

Period	Location (°N, °E)	Abbreviation	Notes	Ref.
Nov 2015	16.85, -24.87	Cape Verde_CVAO	Island; Remote	[9,10]
Aug-Sept 2019	32.26, -64.88	Bermuda_Land	Island; NO_2 & nitrate	[11]
Aug-Sept 2019	32.26, -64.88	Bermuda_Marine	Island; NO_2 & nitrate	[11]
Jul-Aug 2014	34.96, 32.38	Cyprus_HighRH	Island; Soil emission	[12]
Jul-Aug 2014	34.96, 32.38	Cyprus_LowRH	Island; Soil emission	[12]
Jul-Aug 2013	42.97, 9.38	France_Corsica	Island; Ground sources	This study

3 Figures

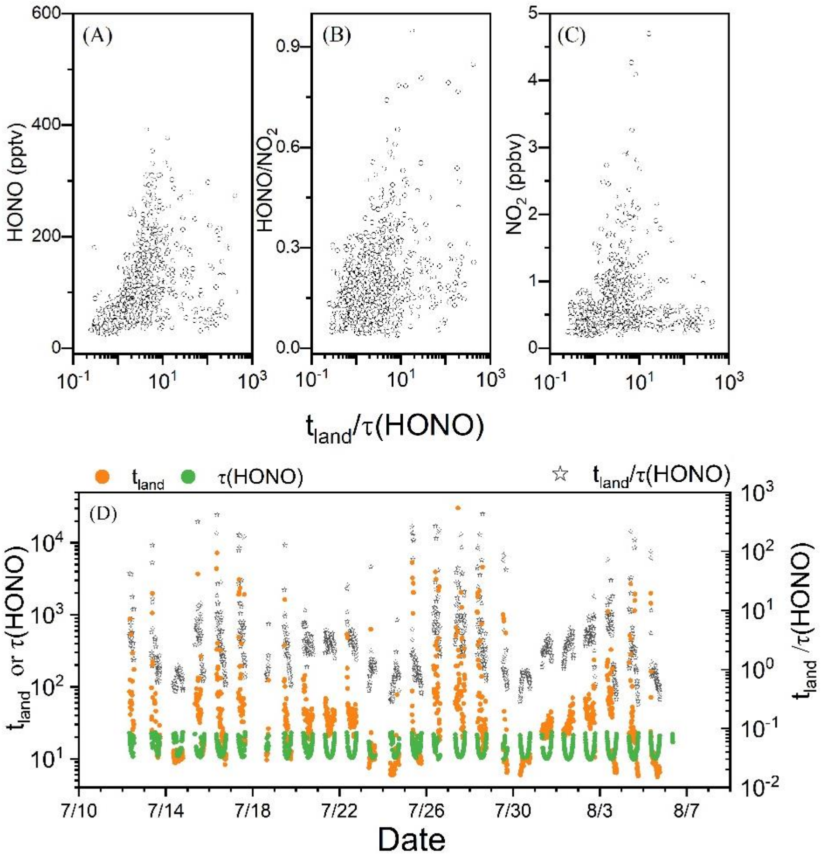

Figure S1. (A) – (C): Daytime HONO, $HONO/NO_2$, and NO_2 plotted against the ratio of the contact time over land (t_{land}) and the lifetime of HONO (τ(HONO)); (D): Time series of t_{land}, τ(HONO), and t_{land}/τ(HONO).

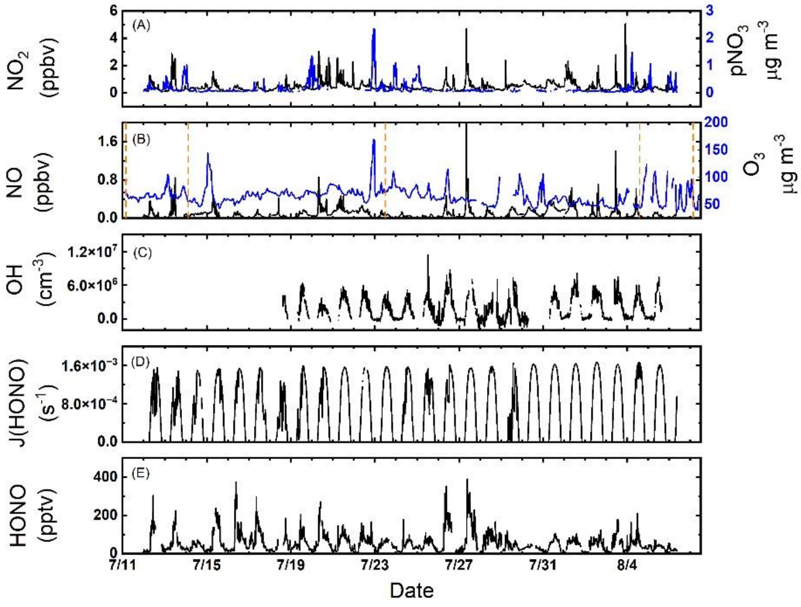

Figure S2. Time series of (A): pNO_3, (B): NO_2, (C): NO, (D): OH, (E): J(HONO), and (F): HONO, and measured during this campaign. The dashed orange lines indicate the occurrence time of potential fire events show in Figure S3.

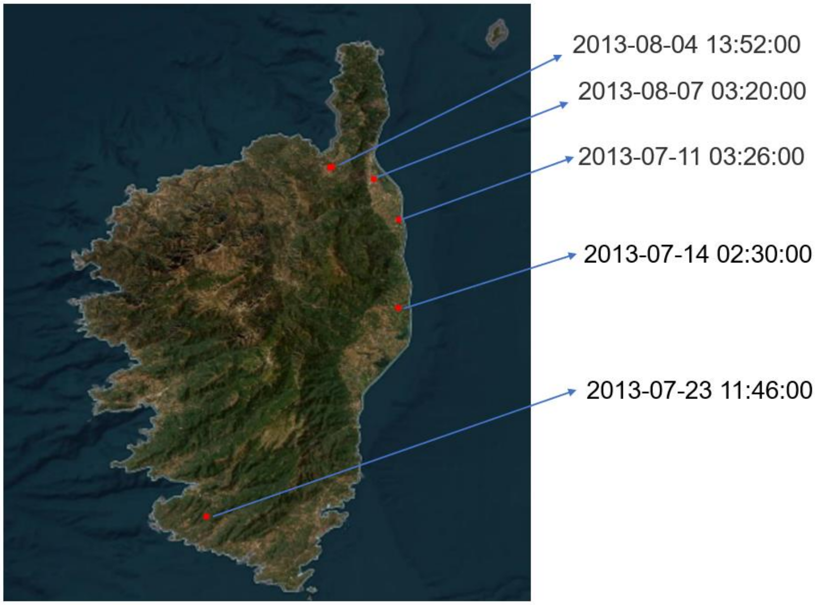

Figure S3. Active fire locations and their occurrent time on Corsica Island during 11 July – 7 August 2013. Data source: https://firms.modaps.eosdis.nasa.gov/. Note that each fire was detected only at the corresponding time on the right panel which indicates the small size and short period of the fire.

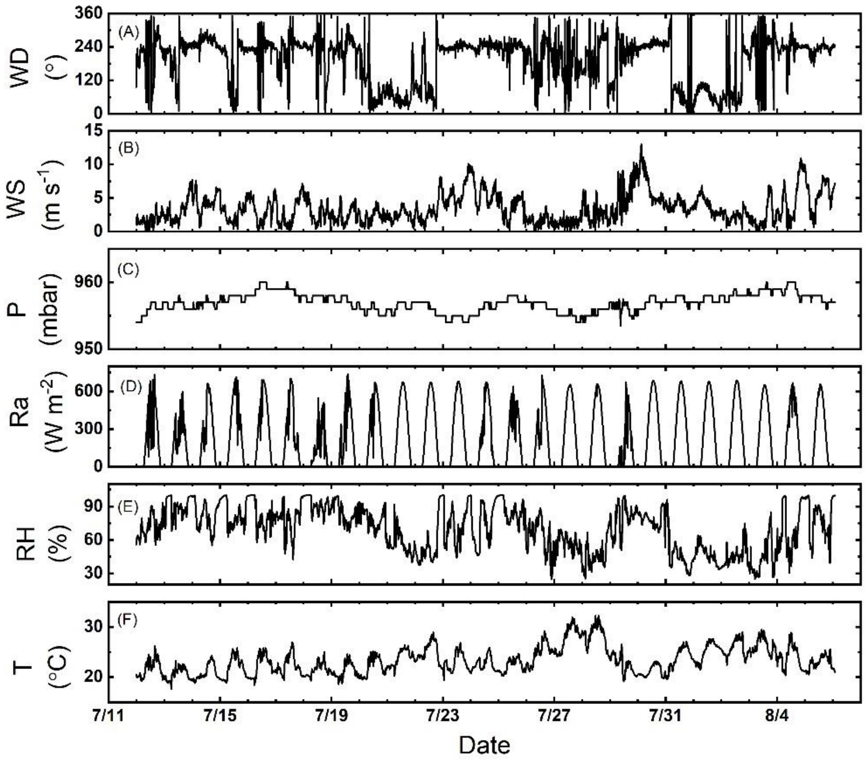

Figure S4. Meteorological measurements during the ChArMEx campaign. (A): wind direction, (B): wind speed, (C): atmospheric pressure, (D): solar irradiance, (E) relative humidity, and (F): temperature.

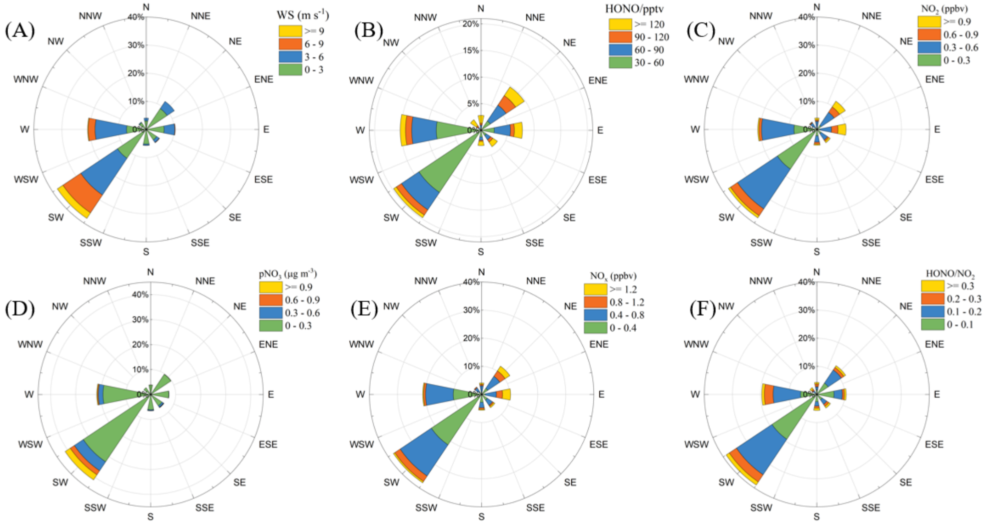

Figure S5. (A): Windrose (wind speed against wind direction) and pollution rose plots (concentration or ratio against wind direction) for (B): HONO, (C): NO_2, (D): pNO_3, (E): NO_x, and (F): HONO/NO_2 during this campaign.

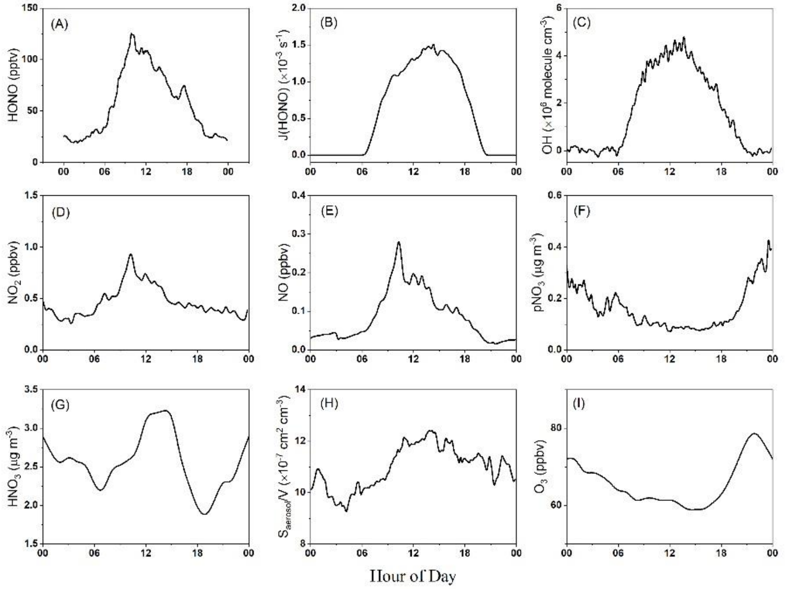

Figure S6. Average diurnal profiles of HONO (A), J(HONO) (B), OH (C), NO_2 (D), NO (E), particulate nitrate (pNO_3) (F), HNO_3 (G), aerosol surface density ($S_{aerosol}/V$) (H) and O_3 (I).

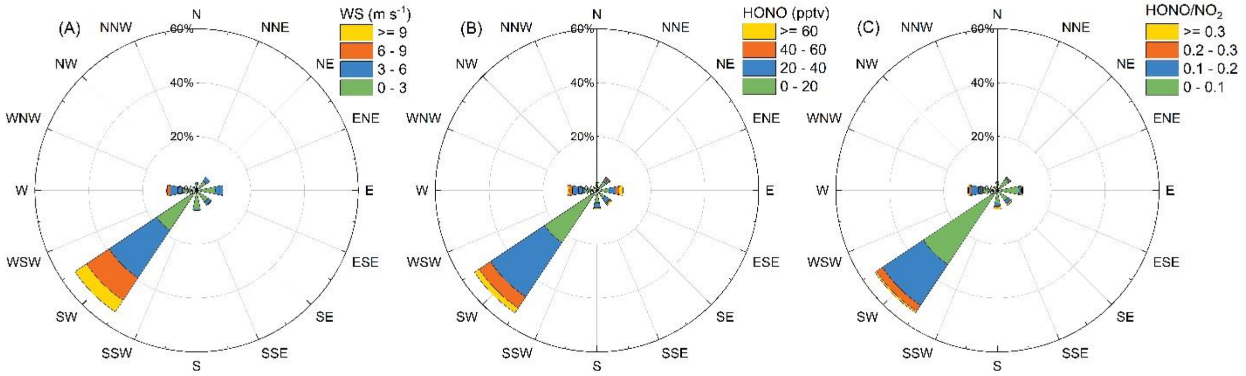

Figure S7. Night-time (A): wind rose and (B): pollution rose plots for HONO and (C): HONO/NO_2.

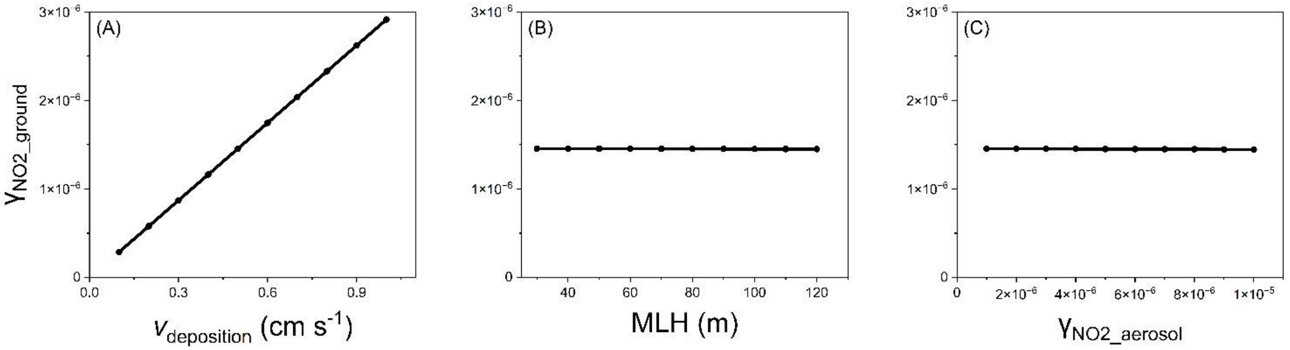

Figure S8. Sensitivities of γ_{NO2_ground} to $v_{deposition}$, MLH, or $\gamma_{NO2\_aerosol}$. For each sensitivity calculation, other parameters remain unchanged in Table 1.

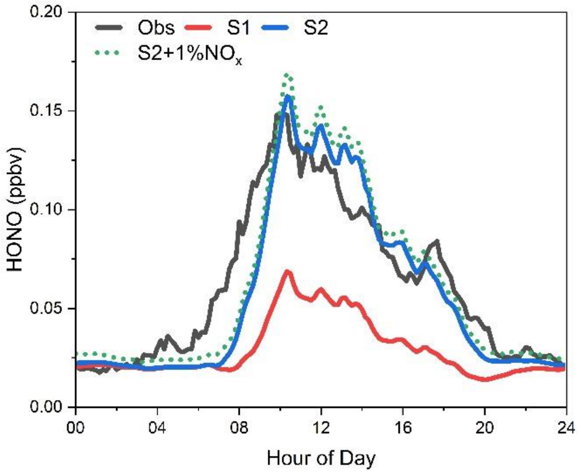

Figure S9. Diurnal profiles of observed and model HONO. Obs: observation; S1: Scenario 1; S2: Scenario 1 with enlarged $\gamma_{NO_2+h\nu\_ground}$ (or with the consideration of SEF impact); S2+1%NO$_x$: S2 with consideration of an assumed HONO-to-NO$_x$ emission ratio of 1% from exhaust of vehicles or ships.

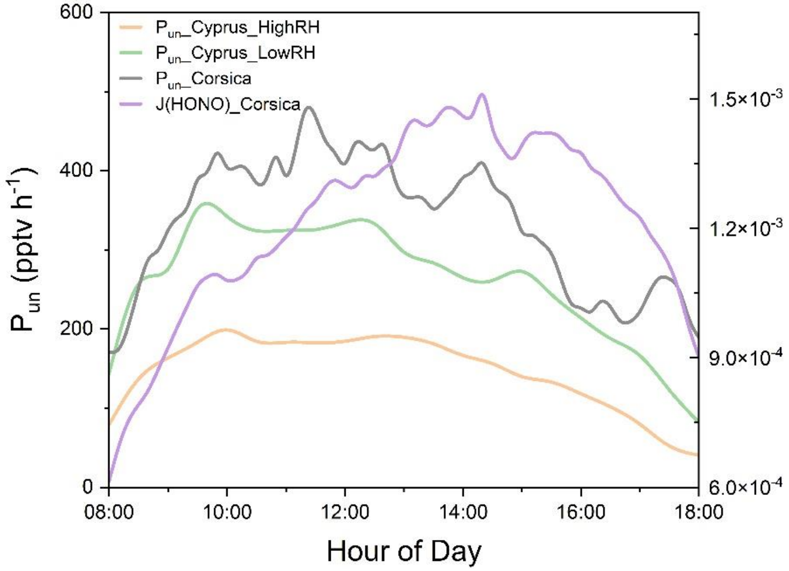

Figure S10. Diurnal profiles of P$_{unknown}$ at Corsica and Cyprus sites.

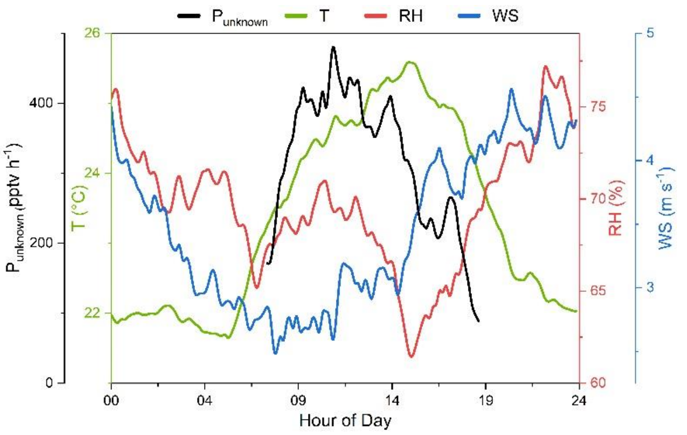

Figure S11. Average diurnal profiles of $P_{unknown}$, atmospheric temperature (T), relative humidity (RH), and wind speed (WS) at the Corsica site.

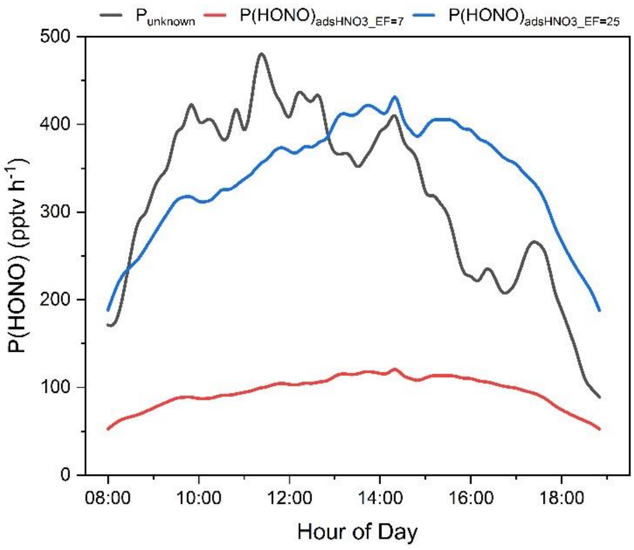

Figure S12. $P_{unknown}$ and the calculated HONO production rate from the photolysis of adsorbed HNO_3.

4 References

(1) Scheer, V.; Frenzel, A.; Behnke, W.; Zetzsch, C.; Magi, L.; George, C.; Mirabel, P. Uptake of Nitrosyl Chloride (NOCl) by Aqueous Solutions. *J. Phys. Chem. A* **1997**, *101* (49), 9359–9366. https://doi.org/10.1021/jp972143m.

(2) Rubasinghege, G.; Grassian, V. H. Surface-Catalyzed Chlorine and Nitrogen Activation: Mechanisms for the Heterogeneous Formation of ClNO, NO, NO2 , HONO, and N2 O from HNO3 and HCl on Aluminum Oxide Particle Surfaces. *J. Phys. Chem. A* **2012**, *116* (21), 5180–5192. https://doi.org/10.1021/jp301488b.

(3) Sander, R. Compilation of Henry's Law Constants (Version 4.0) for Water as Solvent. *Atmos. Chem. Phys.* **2015**, *15* (8), 4399–4981. https://doi.org/10.5194/acp-15-4399-2015.

(4) Wolfe, G. M.; Marvin, M. R.; Roberts, S. J.; Travis, K. R.; Liao, J. The Framework for 0-D Atmospheric Modeling (F0AM) v3.1. *Geosci. Model Dev.* **2016**, *9* (9), 3309–3319. https://doi.org/10.5194/gmd-9-3309-2016.

(5) Xue, C.; Ye, C.; Kleffmann, J.; Zhang, W.; He, X.; Liu, P.; Zhang, C.; Zhao, X.; Liu, C.; Ma, Z.; Liu, J.; Wang, J.; Lu, K.; Catoire, V.; Mellouki, A.; Mu, Y. Atmospheric Measurements at Mt. Tai – Part II: HONO Budget and Radical (RO_x + NO_3) Chemistry in the Lower Boundary Layer. *Atmos. Chem. Phys.* **2022**, *22* (2), 1035–1057. https://doi.org/10.5194/acp-22-1035-2022.

(6) Heland, J.; Kleffmann, J.; Kurtenbach, R.; Wiesen, P. A New Instrument To Measure Gaseous Nitrous Acid (HONO) in the Atmosphere. *Environ. Sci. Technol.* **2001**, *35* (15), 3207–3212. https://doi.org/10.1021/es000303t.

(7) Kukui, A.; Chartier, M.; Wang, J.; Chen, H.; Dusanter, S.; Sauvage, S.; Michoud, V.; Locoge, N.; Gros, V.; Bourrianne, T.; Sellegri, K.; Pichon, J.-M. Role of Criegee Intermediates in the Formation of Sulfuric Acid at a Mediterranean (Cape Corsica) Site under Influence of Biogenic Emissions. *Atmos. Chem. Phys.* **2021**, *21* (17), 13333–13351. https://doi.org/10.5194/acp-21-13333-2021.

(8) Petetin, H.; Sciare, J.; Bressi, M.; Gros, V.; Rosso, A.; Sanchez, O.; Sarda-Estève, R.; Petit, J.-E.; Beekmann, M. Assessing the Ammonium Nitrate Formation Regime in the Paris Megacity and Its Representation in the CHIMERE Model. *Atmos. Chem. Phys.* **2016**, *16* (16), 10419–10440. https://doi.org/10.5194/acp-16-10419-2016.

(9) Crilley, L. R.; Kramer, L. J.; Pope, F. D.; Reed, C.; Lee, J. D.; Carpenter, L. J.; Hollis, L. D. J.; Ball, S. M.; Bloss, W. J. Is the Ocean Surface a Source of Nitrous Acid (HONO) in the Marine Boundary Layer? *Atmos. Chem. Phys.* **2021**, *21* (24), 18213–18225. https://doi.org/10.5194/acp-21-18213-2021.

(10) Reed, C.; Evans, M. J.; Crilley, L. R.; Bloss, W. J.; Sherwen, T.; Read, K. A.; Lee, J. D.; Carpenter, L. J. Evidence for Renoxification in the Tropical Marine Boundary Layer. *Atmos. Chem. Phys.* **2017**, *17* (6), 4081–4092. https://doi.org/10.5194/acp-17-4081-2017.

(11) Zhu, Y.; Wang, Y.; Zhou, X.; Elshorbany, Y. F.; Ye, C.; Hayden, M.; Peters, A. J. An Investigation into the Chemistry of HONO in the Marine Boundary Layer at Tudor Hill Marine Atmospheric Observatory in Bermuda. *Atmos. Chem. Phys.* **2022**, *22* (9), 6327–6346. https://doi.org/10.5194/acp-22-6327-2022.

(12) Meusel, H.; Kuhn, U.; Reiffs, A.; Mallik, C.; Harder, H.; Martinez, M.; Schuladen, J.; Bohn, B.; Parchatka, U.;

Crowley, J. N.; Fischer, H.; Tomsche, L.; Novelli, A.; Hoffmann, T.; Janssen, R. H. H.; Hartogensis, O.; Pikridas, M.; Vrekoussis, M.; Bourtsoukidis, E.; Weber, B.; Lelieveld, J.; Williams, J.; Pöschl, U.; Cheng, Y.; Su, H. Daytime Formation of Nitrous Acid at a Coastal Remote Site in Cyprus Indicating a Common Ground Source of Atmospheric HONO and NO. *Atmos. Chem. Phys.* **2016**, *16* (22), 14475–14493. https://doi.org/10.5194/acp-16-14475-2016.
